# Self-reported snoring is associated with nonalcoholic fatty liver disease

**DOI:** 10.1038/s41598-020-66208-1

**Published:** 2020-06-09

**Authors:** Hui Wang, Qian Gao, Simin He, Yanping Bao, Hongwei Sun, Lingxian Meng, Jie Liang, Chenming Sun, Shuohua Chen, Liying Cao, Wei Huang, Yanmin Zhang, Jianjun Huang, Shouling Wu, Tong Wang

**Affiliations:** 1 Department of Epidemiology and Health Statistics, School of Public Health, Shanxi Medical University, Taiyuan, 030001 China; 2 National Institute on Drug Dependence, Peking University, 38 Xueyuan Road, Beijing, 100191 China; 3 Department of Urology, General Hospital of Datong Coal Mining Group, Datong, 037003 China; 4 Department of Cardiology, Kailuan General Hospital, Tangshan, 063000 China; 5 Department of Hepatobiliary Surgery, Kailuan General Hospital, Tangshan, 063000 China; 6 Department of Ultrasonography, Kailuan General Hospital, Tangshan, 063000 China; 7 Department of Gastroenterology, Kailuan General Hospital, Tangshan, 063000 China; 8 Department of Neurosurgery, General Hospital of Datong Coal Mining Group, Datong, 037003 China

**Keywords:** Non-alcoholic fatty liver disease, Epidemiology, Population screening

## Abstract

Although nonalcoholic fatty liver disease (NAFLD) is associated with obstructive sleep apnea syndrome (OSAS), studies on the direct relationship between NAFLD and snoring, an early symptom of OSAS, are limited. We evaluated whether snorers had higher risk of developing NAFLD. The study was performed using data of the Tongmei study (cross-sectional survey, 2,153 adults) and Kailuan study (ongoing prospective cohort, 19,587 adults). In both studies, NAFLD was diagnosed using ultrasound; snoring frequency was determined at baseline and classified as none, occasional (1 or 2 times/week), or habitual (≥3 times/week). Odds ratios (ORs) and hazard ratios (HRs) with 95% confidence intervals were estimated using logistic and Cox models, respectively. During 10 years’ follow-up in Kailuan, 4,576 individuals with new-onset NAFLD were identified at least twice. After adjusting confounders including physical activity, perceived salt intake, body mass index (BMI), and metabolic syndrome (MetS), multivariate-adjusted ORs and HRs for NAFLD comparing habitual snorers to non-snorers were 1.72 (1.25–2.37) and 1.29 (1.16–1.43), respectively. These associations were greater among lean participants (BMI < 24) and similar across other subgroups (sex, age, MetS, hypertension). Snoring was independently and positively associated with higher prevalence and incidence of NAFLD, indicating that habitual snoring is a useful predictor of NAFLD, particularly in lean individuals.

## Introduction

Nonalcoholic fatty liver disease (NAFLD) is one of the most common chronic liver diseases worldwide^1^. NAFLD affects approximately 25% of the global population^2^, with more than 10% of cases occurring in lean people^1^. The prevalence of NAFLD ranges from 6.3% to 27.0% in Chinese adults^3^ and is increasing at a rate of 0.594% per year^4^. The rising prevalence of NAFLD, in conjunction with the pandemic of obesity and metabolic syndrome (MetS), represent an increasing global public health burden^5^.

Snoring is a common condition that is easily detected by co-sleepers. In a recent study among 10,139 people living in rural areas of northern China, 47.2% of men and 37.8% of women self-reported snoring^6^. Several meta-analyses have revealed that snoring is associated with higher risks of diabetes^7^, gestational diabetes mellitus, pregnancy-induced hypertension and preeclampsia^8^, cardiovascular disease, and all-cause mortality^9,10^. Multiple randomized controlled trials have suggested a possible causal relationship between obstructive sleep apnea syndrome (OSAS) and NAFLD^2,11^. Snoring is an early symptom of OSAS;^12^ however, to our knowledge, few studies have investigated the direct relationships between self-reported snoring and NAFLD. Thus, we conducted the first cross-sectional study and first independent validation cohort study designed to investigate whether individuals who self-reported snoring had a higher prevalence and incidence of NAFLD.

Low-to-moderate alcohol intake may have beneficial effects in patients with NAFLD^13,14^. Conversely, alcohol increases upper airway resistance and snoring^15^. To avoid potentially confounding effects associated with alcohol consumption, the present study focused on individuals who reported that they never drank beer, wine, or spirits.

## Results

**Figure 1:**
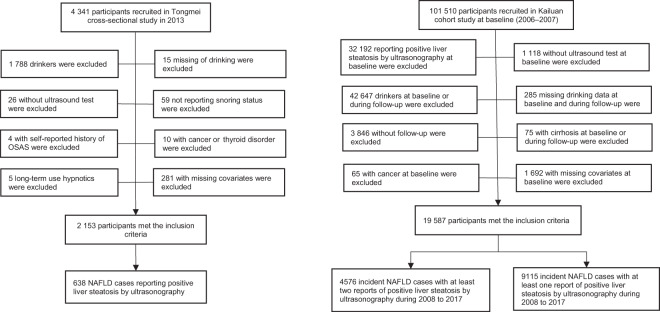
Flow of the selection of study populations, included in Tongmei and Kailuan. NAFLD, nonalcoholic fatty liver disease; OSAS, obstructive sleep apnea syndrome.

In the present study 2,153 participants (mean age 41.4 years) in the Tongmei study and 19,587 participants (mean age 52.7 years) in the Kailuan cohort were included (Fig. 1).

In Tongmei, the prevalence of NAFLD diagnosed via abdominal ultrasound was 29.6% (638/2,153), and those participants were more likely to be men, age ≥ 45 years, and to exhibit higher daily total energy intake, snoring, MetS and its components, higher body mass index (BMI), and elevated alanine transaminase (ALT), aspartate aminotransferase (AST), and gamma glutamyl transpeptidase (GGT). During the 10-year follow-up (follow-up rate 84.8%, 21,422/25,268; calculation is detailed in Supplementary Information), 4,576 patients with incident NAFLD were identified in Kailuan. Those patients were more likely to be women, age 45–65 years, not single, physical labourers, and non-smokers, and they were more likely to work on the surface, have a highest education level of high school, engage in sedentary behaviour for <4 hours per day, have moderate or high perceived salt intake, and exhibit habitual snoring, MetS and its components, higher BMI, and elevated ALT, C-reactive protein (CRP), and serum uric acid (SUA) (Tables 1 and S1).

**Table 1:** Baseline characteristics of participants according to NAFLD status and study (Tongmei and Kailuan).

Characteristic	Tongmei cross-sectional population (2013)				Kailuan cohort at baseline (2006–2007)			
	All^†^ (n = 2153)	Normal liver^‡^ status (n = 1515)	Fatty liver^‡^ status (n = 638)	Crude OR^§^ (95% CI)	All^†^ (n = 19587)	Normal liver^‡^ status (n = 15011)	Fatty liver^‡^ status(n = 4576)	Crude HR(95% CI)
Sex								
Women	533 (24.76%)	414 (77.67%)	119 (22.33%)	1	9176 (46.85%)	6643 (72.40%)	2533 (27.60%)	1
Men	1620 (75.24%)	1101 (67.96%)	519 (32.04%)	**1.64** (**1.30–2.06)**	10411 (53.15%)	8368 (80.38%)	2043 (19.62%)	**0.77** (**0.73–0.82)**
Statistics(P value)		18.14 (<0.0001)				75.37 (<0.0001)		
Age (Years)	41.44 (8.75)	40.76 (8.66)	43.06 (8.75)		52.70 (12.32)	53.07 (12.88)	51.51 (10.19)	
<45	1321 (61.36%)	994 (75.25%)	327 (24.75%)	1	5127 (26.18%)	3973 (77.49%)	1154 (22.51%)	1
45–<55	725 (33.67%)	451 (62.21%)	274 (37.79%)	**1.85** (**1.52–2.25)**	6309 (32.21%)	4507 (71.44%)	1802 (28.56%)	**1.35** (**1.26–1.46)**
55–<65	107 (4.97%)	70 (65.42%)	37 (34.58%)	**1.61** (**1.06–2.44)**	4844 (24.73%)	3666 (75.68%)	1178 (24.32%)	**1.15** (**1.06–1.25)**
≥65	—	—	—	—	3307 (16.88%)	2865 (86.63%)	442 (13.37%)	**0.71** (**0.63–0.79)**
Statistics(P value)		39.49 (<0.0001)				174.75 (<0.0001)		
Marital status								
Single	116 (5.39%)	91 (78.45%)	25 (21.55%)	1	248 (1.27%)	224 (90.32%)	24 (9.68%)	1
Married	1997 (92.75%)	1396 (69.90%)	601 (30.10%)	1.55 (0.98–2.43)	18739 (95.67%)	14292 (76.27%)	4447 (23.73%)	**2.58** (**1.73–3.85)**
Divorced/widowed/separated	40 (1.86%)	28 (70.00%)	12 (30.00%)	1.57 (0.70–3.52)	600 (3.06%)	495 (82.50%)	105 (17.50%)	**2.04** (**1.31–3.18)**
Statistics(P value)		3.84 (0.1466)				26.82 (<0.0001)		
Current tobacco smoking								
No	1196 (55.55%)	845 (70.65%)	351 (29.35%)	1	17255 (88.09%)	13147 (76.19%)	4108 (23.81%)	1
Yes	957 (44.45%)	670 (70.01%)	287 (29.99%)	1.03 (0.86–1.24)	2332 (11.91%)	1864 (79.93%)	468 (20.07%)	**0.89** (**0.81–0.98)**
Statistics(P value)		0.10 (0.7459)				6.11 (0.0134)		
Snoring								
No	879 (40.83%)	707 (80.43%)	172 (19.57%)	1	15128 (77.23%)	11651 (77.02%)	3477 (22.98%)	1
Occasional	774 (35.95%)	553 (71.45%)	221 (28.55%)	**1.64** (**1.31–2.06)**	2944 (15.03%)	2298 (78.06%)	646 (21.94%)	0.97 (0.89–1.05)
Habitual	500 (23.22%)	255 (51.00%)	245 (49.00%)	**3.95** (**3.10–5.03)**	1515 (7.73%)	1062 (70.10%)	453 (29.90%)	**1.41** (**1.27–1.55)**
Statistics(P value)		133.08 (<0.0001)				49.51 (<0.0001)		
MetS								
No	1303 (60.52%)	1118 (85.80%)	185 (14.20%)	1	16737 (85.45%)	13157 (78.61%)	3580 (21.39%)	1
Yes	850 (39.48%)	397 (46.71%)	453 (53.29%)	**6.90** (**5.61–8.47)**	2850 (14.55%)	1854 (65.05%)	996 (34.95%)	**1.95** (**1.81–2.09)**
Statistics(P value)		377.09 (<0.0001)				343.97 (<0.0001)		
Arterial hypertension								
No	1187 (55.13%)	926 (78.01%)	261 (21.99%)	1	9917 (50.63%)	7813 (78.78%)	2104 (21.22%)	1
Yes	966 (44.87%)	589 (60.97%)	377 (39.03%)	**2.27** (**1.88–2.74)**	9670 (49.37%)	7198 (74.44%)	2472 (25.56%)	**1.36** (**1.28–1.44)**
Statistics(P value)		74.15 (<0.0001)				105.49 (<0.0001)		
Hyperglycaemia								
No	1834 (86.02%)	1349 (73.56%)	485 (26.44%)	1	15246 (77.84%)	11715 (76.84%)	3531 (23.16%)	1
Yes	298 (13.98%)	153 (51.34%)	145 (48.66%)	**2.64** (**2.05–3.38)**	4341 (22.16%)	3296 (75.93%)	1045 (24.07%)	**1.12** (**1.05–1.20)**
Statistics(P value)		60.76 (<0.0001)				10.40 (0.0013)		
Hypertriglyceridemia								
No	1385 (64.33%)	1133 (81.81%)	252 (18.19%)	1	15193 (77.57%)	12093 (79.60%)	3100 (20.40%)	1
Yes	768 (35.67%)	382 (49.74%)	386 (50.26%)	**4.54** (**3.73–5.53)**	4394 (22.43%)	2918 (66.41%)	1476 (33.59%)	**1.87** (**1.76–1.99)**
Statistics(P value)		243.61 (<0.0001)				392.99 (<0.0001)		
Low HDL-C								
No	747 (34.70%)	629 (84.20%)	118 (15.80%)	1	17136 (87.49%)	13196 (77.01%)	3940 (22.99%)	1
Yes	1406 (65.30%)	886 (63.02%)	520 (36.98%)	**3.13** (**2.50–3.92)**	2451 (12.51%)	1815 (74.05%)	636 (25.95%)	**1.12** (**1.03–1.21)**
Statistics(P value)		105.02 (<0.0001)				6.52 (0.0107)		
Waist circumference (cm)	88.97 (9.51)	86.02 (8.72)	95.96 (7.43)		83.75 (10.00)	82.86 (10.06)	86.67 (9.20)	
Normal	967 (44.91%)	882 (91.21%)	85 (8.79%)	1	14259 (72.80%)	11495 (80.62%)	2764 (19.38%)	1
Elevated	1186 (55.09%)	633 (53.37%)	553 (46.63%)	**9.07** (**7.06–11.64)**	5328 (27.20%)	3516 (65.99%)	1812 (34.01%)	**2.00** (**1.89–2.13)**
Statistics(P value)		365.73 (<0.0001)				526.93 (<0.0001)		
BMI (kg/m^2^)	24.62 (3.53)	23.27 (2.83)	27.85 (2.85)		23.92 (3.13)	23.45 (3.03)	25.49 (2.97)	
<18	46 (2.14%)	46 (100.00%)	0 (0.00%)	0.25 (0.01–4.25)	319 (1.63%)	312 (97.81%)	7 (2.19%)	**0.15** (**0.07–0.31)**
18–<24	907 (42.13%)	870 (95.92%)	37 (4.08%)	1	10026 (51.19%)	8610 (85.88%)	1416 (14.12%)	1
24–<28	833 (38.69%)	521 (62.55%)	312 (37.45%)	**13.91** (**9.74–19.86)**	7444 (38.00%)	5111 (68.66%)	2333 (31.34%)	**2.59** (**2.42–2.77)**
≥28	367 (17.05%)	78 (21.25%)	289 (78.75%)	**85.61** (**56.73–129.19)**	1798 (9.18%)	978 (54.39%)	820 (45.61%)	**4.37** (**4.01–4.77)**
Statistics(P value)		752.39 (<0.0001)				1365.82(<0.0001)		
Obesity								
Normal	694 (32.23%)	679 (97.84%)	15 (2.16%)	1	8904 (45.46%)	7780 (87.38%)	1124 (12.62%)	1
Simple central obesity	259 (12.03%)	237 (91.51%)	22 (8.49%)	**4.20** (**2.14–8.23)**	1441 (7.36%)	1142 (79.25%)	299 (20.75%)	**1.73** (**1.53–1.97)**
Simple overweight	273 (12.68%)	203 (74.36%)	70 (25.64%)	**15.61** (**8.74–27.85)**	5355 (27.34%)	3715 (69.37%)	1640 (30.63%)	**2.83** (**2.62–3.05)**
Both forms of obesity	927 (43.06%)	396 (42.72%)	531 (57.28%)	**60.69** (**35.80–102.88)**	3887 (19.84%)	2374 (61.08%)	1513 (38.92%)	**3.94** (**3.65–4.26)**
Statistics(P value)		648.61 (<0.0001)				1302.34 (<0.0001)		

^†^Values are displayed as number (column percent) for categorical variables and mean (SD) for continuous variables.

^‡^Values are displayed as number (row percent) for categorical variables and mean (SD) for continuous variables.

^§^Logistic regression was used for calculating crude ORs in univariate analyses; The ORs according to BMI were calculated using Firth’s penalized likelihood because of quasi-complete separation of data points.

Cox regression was used for calculating crude HRs in univariate analyses.

Abbreviation. CI confidence interval; OR odds ratio; HR, hazard ratio; SD, standard deviation; MetS, metabolic syndrome; HDL-C, high-density lipoprotein cholesterol; BMI, body mass index; WC, waist circumference; NAFLD, nonalcoholic fatty liver disease.

Compared with non-snorers, all snorers had higher prevalence and incidence of NAFLD, after adjusting for potential confounders; however, this was not the case for occasional snorers. Consistent results were obtained in sensitivity analyses (Table 2).

**Table 2:** Effect of self-reported snoring status on NAFLD after adjusting covariates in Tongmei and Kailuan.

	Tongmei cross-sectional population in 2013 (N = 2153)^‡^				Kailuan cohort from 2006 to 2017 (N = 19587))^§^			
	Non-snorers	Occasional	Habitual	Snorers	Non-snorers	Occasional	Habitual	Snorers
NAFLD^*^								
Cases/total (n)	172/879	221/774	245/500	466/1274	3477/15128	646/2944	453/1515	1099/4459
Incidence rate, per 1000 PYs	—	—	—	—	31.9	30.5	45.0	35.2
Model1^†^	1	1.15 (0.85–1.55) P = 0.3644	**1.72 (1.25–2.37) P** = **0.0009**	**1.37 (1.05–1.79) P** = **0.0203**	1	1.01 (0.92–1.11) P = 0.8365	**1.29 (1.16–1.43) P** = < 0**.0001**	**1.10 (1.02–1.19) P** = **0.0118**
**Sensitivity analysis**								
Cases/total (n)	172/879	221/774	245/500	466/1274	6959/15128	1338/2944	826/1515	2164/4459
Incidence rate, per 1000 PYs	—	—	—	—	67.6	68.7	90.0	75.5
Model2^†^	1	1.08 (0.94–1.24) P = 0.2753	**1.25 (1.08–1.43) P** = **0.0023**	**1.16 (1.02–1.31) P** = **0.0233**	1	1.04 (0.97–1.11) P = 0.2560	**1.24 (1.14–1.34) P** = < **.0001**	**1.10 (1.04–1.16) P** = **0.0004**

^*^NAFLD cases were defined as having positive ultrasonography results, and incident cases were those without NAFLD at baseline and with at least two positive ultrasonography results during 2008–2017.

^†^Logistic regression was used in Model 1 and Poisson regression was used in Model 2 in Tongmei. Cox proportional hazards regression was used in Kailuan. The reference level was non-snorers in all models. Snorers included occasional snorers and habitual snorers. ORs (95% CIs) were estimated in the logistic regression, HRs (95% CIs) were estimated in Cox proportional regression, and RRs (95% CIs) were estimated in Poisson regression.

^‡^ adjusted for age (<45, ≥45 years), sex, marital status (single, married, divorced/widowed/separated), education (illiterate/primary, junior high school, senior high school or college, bachelor’s degree or higher), income (≤4000, >4000–6000, >6000 RMB), workplace (underground/surface), occupation type (mental labour/light physical labour/heavy physical labour), current tobacco smoking (yes, no), perceived salt intake (low, medium, high), degree of IPAQ (low, moderate, high), degree of sedentary (low, moderate, high), total energy intake per day (low, moderate, high), elevated serum liver enzymes (no/yes), obesity (normal, simple central, simple overweight, both), BMI (<24, 24–<28, ≥28 kg/m^2^), and MetS (no/yes) in Tongmei.

^§^ adjusted for age (<45, 45–<55, 55–<65, ≥65 years), sex, marital status (single, married, divorced/widowed/separated), education (illiterate/primary, junior high school, senior high school, college or higher), income (<600, 600–800, 800–1000, >1000 RMB), workplace (underground/surface), occupation type (mental labour/physical labour), smoking status (never, former, current), perceived salt intake (low, medium, high), physical activity (no, occasional, always), sedentary duration (<4, 4–8, >8 hours per day), elevated ALT (>40 U/L), obesity (normal, simple central, simple overweight, both), elevated SUA (>357μmol/ L for women and >420μmol/ L for men), CRP (<1, 1–3, >3 mg/L), BMI (<18, 18–<24, 24–<28, ≥28 kg/m^2^), and MetS (no/yes) in Kailuan.

NAFLD cases were defined as having positive ultrasonography results, and incident cases were those without NAFLD at baseline and with at least one positive ultrasonography result during 2008–2017. Abbreviation: NAFLD, non–alcoholic fatty liver disease; CI, confidence interval; OR, odds ratio; HR, hazard ratio; RR relative risk; BMI, body mass index; MetS, metabolic syndrome; IPAQ, international physical activity questionnaire; ALT, alanine transaminase; SUA, serum uric acid; CRP, C–reactive protein; PYs, person-years.

**Figure 2:**
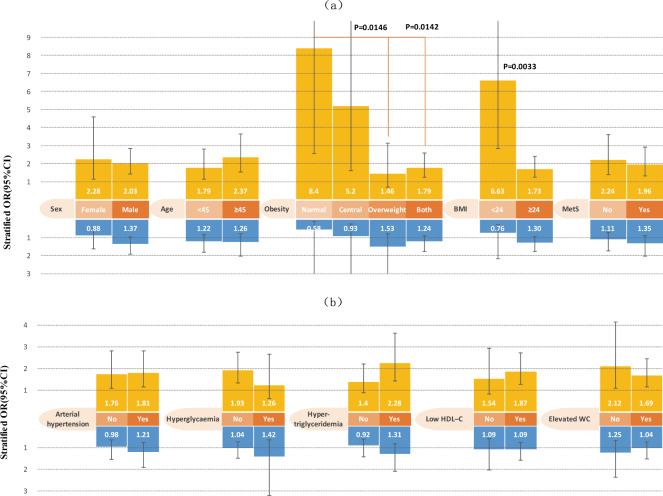
Stratified odds ratio (OR) (95% confidence interval (CI)) of snoring on nonalcoholic fatty liver disease according to (**a**) age, sex, obesity, body mass index(BMI) and metabolic syndrome (MetS), and (**b**) MetS components in the Tongmei population, adjusted for age (<45 or ≥45 years), sex, marital status (single, married, divorced/widowed/separated), education (illiterate/primary, junior high school, senior high school or college, bachelor’s degree or higher), income (≤4000, >4000–6000, >6000 RMB), workplace (underground/surface), occupation type (mental labour/light physical labour/heavy physical labour), current tobacco smoking (yes, no), perceived salt intake (low, medium, high), degree of International physical activity questionnaire (IPAQ) (low, moderate, high), degree of sedentary behaviour (low, moderate, high), total energy intake per day (low, moderate, high), elevated serum liver enzymes (no/yes), obesity (normal, central, overweight, both), and MetS (no/yes). Yellow indicates habitual snorers compared with non-snorers; blue indicates occasional snorers compared with non-snorers. Significant P values are shown for interaction on a multiplicative scale.

In Tongmei, habitual snoring was still associated with the prevalence of NAFLD in each stratum after stratifying by sex, age (<45 vs. ≥45 years), workplace (underground vs. surface), occupation type, BMI, MetS, arterial hypertension, and waist circumference (WC); however, this was only significant in participants who did not have simple overweight or hyperglycaemia, and those who had hypertriglyceridemia or low high-density lipoprotein cholesterol (HDL-C). Obesity, BMI, and hypertriglyceridemia modified the effects of habitual snoring on NAFLD (Fig. 2, Tables 3 and S2). The association between habitual snoring and NAFLD prevalence was stronger among lean participants (BMI < 24 or those with normal BMI and WC), and patients with hypertriglyceridemia.

**Table 3:** Effect modification of snoring on NAFLD in Tongmei: OR (95% CI), P value.

Variables	Snoring	Occasional snorers (N = 774) vs. Non–snorers (N = 879)			Habitual snorers (N = 500) vs. Non–snorers (N = 879)		
		NAFLD (No/Yes)	OR (95% CI), P value	Stratified OR (95% CI), P value	NAFLD (No/Yes)	OR (95% CI), P value	Stratified OR (95% CI), P value
Sex^†^							
Women	No	224/44	1 (ref)	1 (ref)	224/44	1 (ref)	1 (ref)
Women	Yes	148/42	0.88 (0.48–1.63), P = 0.6909	0.88 (0.48–1.63), P = 0.6909	42/33	**2.28 (1.13–4.60), P** = **0.0218**	**2.28** (**1.13–4.60), P** = **0.0218**
Men	No	483/128	0.58 (0.32–1.06), P = 0.0751	1 (ref)	483/128	**0.48** (**0.27–0.86), P** = **0.0136**	1 (ref)
Men	Yes	405/179	0.80 (0.44–1.44), P = 0.4528	1.37 (0.98–1.92), P = 0.0687	213/212	0.97 (0.54–1.76), P = 0.9313	**2.03** (**1.44–2.86), P** < 0**.0001**
Ratio of ORs			1.55 (0.77–3.12), P = 0.2168			0.89 (0.41–1.94), P = 0.7718	
RERI			0.33 (–0.21~0.87), P = 0.2258			–0.78 (–2.30~0.74), P = 0.3136	
Age (Year)^†^							
<45	No	474/88	1 (ref)	1 (ref)	474/88	1 (ref)	1 (ref)
<45	Yes	395/135	1.22 (0.83–1.79), P = 0.3066	1.22 (0.83–1.79), P = 0.3066	125/104	**1.79** (**1.15–2.80), P** = **0.0102**	**1.79** (**1.15–2.80), P** = **0.0102**
≥45	No	233/84	1.22 (0.78–1.93), P = 0.3823	1 (ref)	233/84	1.39 (0.89–2.18), P = 0.1460	1 (ref)
≥45	Yes	158/86	1.55 (0.97–2.48), P = 0.0688	1.26 (0.79–2.03), P = 0.3305	130/141	**3.30** (**2.13–5.12), P** < 0**.0001**	**2.37** (**1.54–3.63), P** < 0**.0001**
Ratio of ORs			1.03 (0.56–1.90), P = 0.9123			1.32 (0.72–2.44), P = 0.3713	
RERI			0.10 (–0.68~0.88), P = 0.7975			1.12 (–0.12~2.35), P = 0.0770	
Obesity^†^							
Normal	No	368/5	1 (ref)	1 (ref)	368/5	1 (ref)	1 (ref)
Normal	Yes	249/2	0.58 (0.11–3.05), P = 0.5216	0.58 (0.11–3.05), P = 0.5216	62/8	**8.40** (**2.57–27.43), P** = **0.0004**	**8.40** (**2.57–27.43), P** = **0.0004**
Simple central	No	111/5	2.75 (0.77–9.87), P = 0.1198	1 (ref)	111/5	2.71 (0.75–9.74), P = 0.1278	1 (ref)
Simple central	Yes	91/5	2.55 (0.65–9.96), P = 0.1771	0.93 (0.24–3.64), P = 0.9139	35/12	**14.07** (**4.41–44.91), P** < 0**.0001**	**5.20** (**1.62–16.71), P** = **0.0057**
Simple overweight	No	87/21	**16.35** (**5.91–45.26), P** < 0**.0001**	1 (ref)	87/21	**17.02** (**6.14–47.17), P** < 0**.0001**	1 (ref)
Simple overweight	Yes	66/29	**25.06** (**9.17–68.50), P** < 0**.0001**	1.53 (0.77–3.05), P = 0.2250	50/20	**24.92** (**8.68–71.52), P** < 0**.0001**	1.46 (0.69–3.12), P = 0.3250
Both	No	141/141	**43.22** (**16.93–110.31), P** < 0**.0001**	1 (ref)	141/141	**45.55** (**17.79–116.63), P** < 0**.0001**	1 (ref)
Both	Yes	147/185	**53.72** (**21.12–136.61), P** < 0**.0001**	1.24 (0.88–1.76), P = 0.2214	108/205	**81.51** (**31.52–210.76), P** < 0**.0001**	**1.79** (**1.24–2.58), P** = **0.0018**
Ratio of ORs			1.59 (0.19–13.67), P = 0.6709	Central vs normal		0.62 (0.12–3.25), P = 0.5708	Central vs normal
Ratio of ORs			2.63 (0.44–15.82), P = 0.2900	Overweight vs normal		**0.17** (**0.04–0.71), P** = **0.0146**	Overweight vs normal
Ratio of ORs			2.14 (0.39–11.60), P = 0.3793	Both vs normal		**0.21** (**0.06–0.73), P** = **0.0142**	Both vs normal
RERI			0.22 (–3.56~3.99), P = 0.9099	Central vs normal		3.96 (–8.76~16.69), P = 0.5415	Central vs normal
RERI			9.12 (–6.50~24.74), P = 0.2525	Overweight vs normal		0.49 (–16.86~17.84), P = 0.9556	Overweight vs normal
RERI			10.92 (–8.08~29.91), P = 0.2599	Both vs normal		28.56 (–6.06~63.18), P = 0.1058	Both vs normal
BMI (Kg/m^2^)^†^							
<24	No	479/10	1 (ref)	1 (ref)	479/10	1 (ref)	1 (ref)
<24	Yes	340/7	0.76 (0.27–2.16), P = 0.6086	0.76 (0.27–2.16), P = 0.6086	97/20	**6.63** (**2.86–15.37), P** < 0**.0001**	**6.63** (**2.86–15.37), P** < 0**.0001**
≥24	No	228/162	**45.93** (**19.07–110.59), P** < 0**.0001**	1 (ref)	228/162	**40.84** (**18.66–89.38), P** < 0**.0001**	1 (ref)
≥24	Yes	213/214	**59.55** (**24.79–143.04), P** < 0**.0001**	1.30 (0.95–1.77), P = 0.1034	158/225	**70.48** (**31.79–156.26), P** < 0**.0001**	**1.73** (**1.24–2.40), P** = **0.0012**
Ratio of ORs			1.70 (0.57–5.05), P = 0.3378			**0.26** (**0.11–0.64), P** = **0.0033**	
RERI			13.86 (–6.14~33.86), P = 0.1743			24.01 (–3.87~51.88), P = 0.0915	
MetS^†^							
No	No	555/61	1 (ref)	1 (ref)	555/61	1 (ref)	1 (ref)
No	Yes	408/64	1.11 (0.71–1.73), P = 0.6569	1.11 (0.71–1.73), P = 0.6569	155/60	**2.24** (**1.39–3.61), P** = **0.0009**	**2.24** (**1.39–3.61), P** = **0.0009**
Yes	No	152/111	**2.22** (**1.41–3.49), P** = **0.0005**	1 (ref)	152/111	**2.39** (**1.53–3.74), P** = **0.0001**	1 (ref)
Yes	Yes	145/157	**3.00** (**1.94–4.64), P** < 0**.0001**	1.35 (0.91–2.01), P = 0.1366	100/185	**4.70** (**2.99–7.38), P** < 0**.0001**	**1.96** (**1.32–2.93), P** = **0.0010**
Ratio of ORs			1.22 (0.67–2.22), P = 0.5115			0.88 (0.47–1.62), P = 0.6773	
RERI			0.68 (–0.42~1.77), P = 0.2281			1.07 (–0.64~2.77), P = 0.2192	

^†^adjusted for age (<45 or ≥45 years), sex, marital status (single, married, divorced/widowed/separated), education (illiterate/primary, junior high school, senior high school or college, bachelor or higher), income (≤4000, >4000–6000, >6000 RMB), workplace (underground/surface), occupation type (mental labour/light physical labour/heavy physical labour), current tobacco smoking (yes, no), perceived salt intake (low, medium, high), degree of IPAQ (low, moderate, high), degree of sedentary (low, moderate, high), total energy intake per day (low, moderate, high), elevated serum liver enzymes (no/yes), obesity (normal, central, overweight, both), and MetS (no/yes).

Abbreviation. CI confidence interval; OR odds ratio; HR, hazard ratio; SD, standard deviation; IPAQ, international physical activity questionnaire; MetS, metabolic syndrome; BMI, body mass index; RERI, relative excess risk due to interaction.

**Figure 3:**
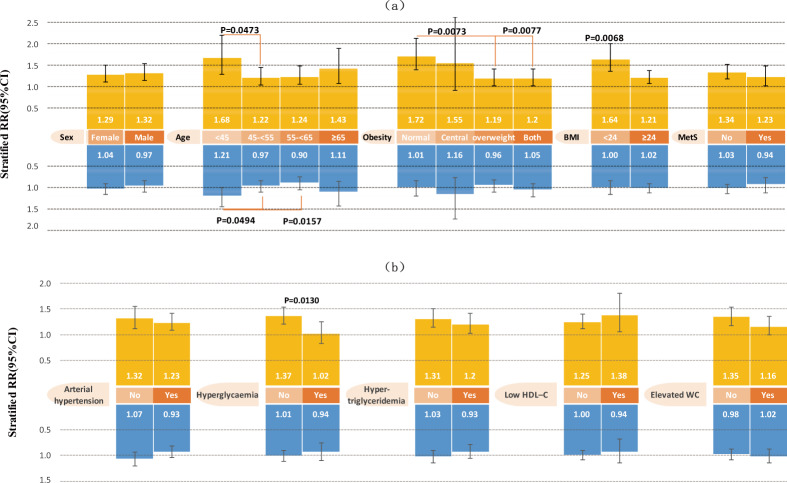
Stratified relative risk (RR) (95% confidence interval (CI)) of snoring on nonalcoholic fatty liver disease according to (**a**) age, sex, obesity, body mass index (BMI) and metabolic syndrome (MetS), and (**b**) MetS components in the Kailuan cohort, adjusted for age (<45, 45–<55, 55–<65, ≥65 years), sex, marital status (single, married, divorced/widowed/separated), education (illiterate/primary, junior high school, senior high school, college or higher), income (<600, 600–800, 800–1000, >1000 RMB), workplace (underground/surface), occupation type (mental labour/physical labour), current tobacco smoking (yes, no), perceived salt intake (low, medium, high), physical activity (no, occasional, always), sedentary duration (<4, 4–8, >8 hours per day), elevated ALT (>40 U/L), obesity (normal, simple central, simple overweight, both), elevated SUA (>357 μmol/ L for women and >420 μmol/ L for men), CRP (<1, 1–3, >3 mg/L), and MetS (no/yes). Yellow indicates habitual snorers compared with non-snorers; blue indicates occasional snorers compared with non-snorers. Significant P values are shown for interaction on a multiplicative scale.

In Kailuan, habitual snoring was still associated with the incidence of NAFLD in each stratum according to sex, age, occupation type, BMI, MetS, arterial hypertension, hypertriglyceridemia, and HDL-C; this was only significant in participants who were surface workers, did not have simple central obesity, or hyperglycaemia, and who had normal WC. Obesity, BMI, and hyperglycaemia modified the effects of habitual snoring on NAFLD (Fig. 3, Table 4 and S3). The association between habitual snoring and NAFLD risk was greater among lean participants (BMI < 24 or those with normal BMI and WC), and those who did not exhibit hyperglycaemia.

**Table 4:** Effect modification of snoring on NAFLD in Kailuan: HR (95% CI), P value.

Variables	Snoring	Occasional snorers (N = 2944) vs. Non-snorers (N = 15128)			Habitual snorers (N = 1515) vs. Non-snorers (N = 15128)		
		NAFLD (No/Yes)	HR (95% CI), P value	Stratified HR (95% CI), P value	NAFLD (No/Yes)	HR (95% CI), P value	Stratified HR (95% CI), P value
Sex^†^							
Women	No	5233/1956	1 (ref)	1 (ref)	5233/1956	1 (ref)	1 (ref)
Women	Yes	1076/380	1.04 (0.92–1.17), P = 0.5258	1.04 (0.92–1.17), P = 0.5258	334/197	**1.29** (**1.10–1.50), P** = **0.0012**	**1.29** (**1.10–1.50), P** = **0.0012**
Men	No	6418/1521	**0.71** (**0.65–0.77), P** < 0**.0001**	1 (ref)	6418/1521	**0.71** (**0.65–0.76), P** < 0**.0001**	1 (ref)
Men	Yes	1222/266	**0.69** (**0.59–0.80),P** < 0**.0001**	0.97 (0.85–1.12), P = 0.6904	728/256	0.93 (0.80–1.09), P = 0.3740	**1.32** (**1.14–1.53), P** = **0.0002**
Ratio of HRs			0.94 (0.79–1.12), P = 0.4628			1.03 (0.84–1.26), P = 0.8031	
RERI			−0.06 (−0.21~0.09), P = 0.4471			−0.06 (−0.29~0.17), P = 0.6027	
Age (Year)^†^							
<45	No	3371/953	1 (ref)	1 (ref)	3371/953	1 (ref)	1 (ref)
<45	Yes	500/144	**1.21** (**1.01–1.45), P** = **0.0387**	**1.21** (**1.01–1.45), P** = **0.0387**	102/57	**1.68** (**1.28–2.20), P** = **0.0002**	**1.68** (**1.28–2.20), P** = **0.0002**
45–<55	No	3489/1388	**1.17** (**1.07–1.27), P** = **0.0004**	1 (ref)	3489/1388	**1.16** (**1.06–1.26), P** = **0.0010**	1 (ref)
45–<55	Yes	697/248	1.13 (0.98–1.30), P = 0.1057	0.97 (0.84–1.11), P = 0.6395	321/166	**1.41** (**1.19–1.68), P** < 0**.0001**	**1.22** (**1.03–1.44), P** = **0.0194**
55–<65	No	2702/833	1.00 (0.91–1.11), P = 0.9707	1 (ref)	2702/833	0.99 (0.89–1.09), P = 0.7790	1 (ref)
55–<65	Yes	599/174	0.90 (0.75–1.07), P = 0.2207	0.90 (0.76–1.06), P = 0.2025	365/171	**1.23** (**1.03–1.46), P** = **0.0240**	**1.24** (**1.05–1.48), P** = **0.0126**
≥65	No	2089/303	**0.62** (**0.54–0.71), P** < 0**.0001**	1 (ref)	2089/303	**0.62** (**0.54–0.71), P** < **.0001**	1 (ref)
≥65	Yes	502/80	**0.69** (**0.54–0.88), P** = **0.0025**	1.11 (0.87–1.43), P = 0.4030	274/59	0.88 (0.67–1.16), P = 0.3674	**1.43** (**1.07–1.90), P** = **0.0141**
Ratio of HRs			**0.80** (**0.64–1.00), P** = **0.0494**	45–<55 vs <45		**0.73** (**0.53–1.00), P** = **0.0473**	45–<55 vs <45
Ratio of HRs			**0.74** (**0.58–0.95), P** = **0.0157**	55–<65 vs <45		0.74 (0.54–1.02), P = 0.0645	55–<65 vs <45
Ratio of HRs			0.92 (0.68–1.25), P = 0.5948	≥ 65 vs <45		0.85 (0.58–1.26), P = 0.4196	≥ 65 vs <45
RERI			−0.25 (−0.51~0.02), P = 0.0660	45–<55 vs <45		−0.42 (−0.92~0.08), P = 0.0982	45–<55 vs <45
RERI			**−0.31** (**−0.58~0.05), P** = **0.0205**	55–<65 vs <45		−0.44 (−0.93~0.05), P = 0.0807	55–<65 vs <45
RERI			−0.14 (−0.41~0.13), P = 0.3169	≥ 65 vs <45		−0.41 (−0.92~0.09), P = 0.1111	≥ 65 vs <45
Obesity^†^							
Normal	No	6086/857	1 (ref)	1 (ref)	6086/857	1 (ref)	1 (ref)
Normal	Yes	1240/165	1.01 (0.85–1.20), P = 0.9100	1.01 (0.85–1.20), P = 0.9100	454/102	**1.72** (**1.39–2.12), P** < 0**.0001**	**1.72** (**1.39–2.12), P** < 0**.0001**
Simple central	No	1004/258	**1.53** (**1.33–1.76), P** < 0**.0001**	1 (ref)	1004/258	**1.51** (**1.31–1.74), P** < 0**.0001**	1 (ref)
Simple central	Yes	99/26	**1.77** (**1.20–2.62), P** = **0.0043**	1.16 (0.77–1.74), P = 0.4762	39/15	**2.34** (**1.40–3.92), P** = **0.0012**	1.55 (0.92–2.62), P = 0.1006
Simple overweight	No	2832/1264	**2.87** (**2.63–3.13), P** < 0**.0001**	1 (ref)	2832/1264	**2.86** (**2.62–3.12), P** < 0**.0001**	1 (ref)
Simple overweight	Yes	562/219	**2.74** (**2.35–3.19), P** < 0**.0001**	0.96 (0.82–1.11), P = 0.5500	321/157	**3.41** (**2.86–4.07), P** < 0**.0001**	**1.19** (**1.01–1.42), P** = **0.0429**
Both	No	1729/1098	**3.61** (**3.27–3.98), P** < 0**.0001**	1 (ref)	1729/1098	**3.56** (**3.22–3.92), P** < 0**.0001**	1 (ref)
Both	Yes	397/236	**3.80** (**3.25–4.43), P** < 0**.0001**	1.05 (0.91–1.22), P = 0.4901	248/179	**4.28** (**3.59–5.10), P** < 0**.0001**	**1.20** (**1.02–1.42), P** = **0.0283**
Ratio of HRs			1.15 (0.74–1.78), P = 0.5371	Central vs normal		0.90 (0.52–1.59), P = 0.7261	Central vs normal
Ratio of HRs			0.95 (0.76–1.18), P = 0.6237	Overweight vs normal		**0.70** (**0.53–0.91), P** = **0.0073**	Overweight vs normal
Ratio of HRs			1.04 (0.84–1.30), P = 0.7080	Both vs normal		**0.70** (**0.54–0.91), P** = **0.0077**	Both vs normal
RERI			0.23 (−0.49~0.96), P = 0.5295	Central vs normal		0.12 (−1.13~1.37), P = 0.8543	Central vs normal
RERI			−0.14 (−0.58~0.30), P = 0.5404	Overweight vs normal		−0.16 (−0.82~0.50), P = 0.6333	Overweight vs normal
RERI			0.18 (−0.39~0.75), P = 0.5328	Both vs normal		0.01 (−0.75~0.77), P = 0.9827	Both vs normal
BMI (Kg/m^2^)^†^							
<24	No	7090/1115	1 (ref)	1 (ref)	7090/1115	1 (ref)	1 (ref)
<24	Yes	1339/191	1.00 (0.85–1.17), P = 0.9776	1.00 (0.85–1.17), P = 0.9776	493/117	**1.64** (**1.35–2.00), P** < 0**.0001**	**1.64** (**1.35–2.00), P** < 0**.0001**
≥24	No	4561/2362	**2.84** (**2.64–3.06), P** < 0**.0001**	1 (ref)	4561/2362	**2.83** (**2.63–3.05), P** < 0**.0001**	1 (ref)
≥24	Yes	959/455	**2.89** (**2.57–3.25), P** < 0**.0001**	1.02 (0.91–1.13), P = 0.7749	569/336	**3.42** (**3.00–3.91), P** < 0**.0001**	**1.21** (**1.07–1.37), P** = **0.0027**
Ratio of HRs			1.02 (0.85–1.22), P = 0.8481			**0.73** (**0.59–0.92), P** = **0.0068**	
RERI			0.05 (−0.29~0.38), P = 0.7832			−0.06 (−0.56~0.45), P = 0.8291	
MetS^†^							
No	No	10335/2764	1 (ref)	1 (ref)	10335/2764	1 (ref)	1 (ref)
No	Yes	1973/505	1.03 (0.93–1.14), P = 0.5478	1.03 (0.93–1.14), P = 0.5478	849/311	**1.34** (**1.18–1.51), P** < 0**.0001**	**1.34** (**1.18–1.51), P** < 0**.0001**
Yes	No	1316/713	**1.31** (**1.19–1.44), P** < 0**.0001**	1 (ref)	1316/713	**1.34** (**1.22–1.47), P** < 0**.0001**	1 (ref)
Yes	Yes	325/141	**1.23** (**1.03–1.47), P** = **0.0249**	0.94 (0.78–1.13), P = 0.5069	213/142	**1.65** (**1.38–1.98), P** < 0**.0001**	**1.23** (**1.02–1.49), P** = **0.0275**
Ratio of HRs			0.91 (0.74–1.12), P = 0.3685			0.92 (0.74–1.15), P = 0.4707	
RERI			−0.11 (−0.36~0.14), P = 0.3761			−0.02 (−0.35~0.31), P = 0.8935	

^†^Adjusted for age (<45, 45–<55, 55–<65, ≥65 years), sex, marital status (single, married, divorced/widowed/separated), education (illiterate/primary, junior high school, senior high school, college or higher), income (<600, 600–800, 800–1000, >1000 RMB), workplace (underground/surface), occupation type (mental labour/physical labour), current tobacco smoking (yes, no), perceived salt intake (low, medium, high), physical activity (no, occasional, always), sedentary duration (<4, 4–8, >8 hours per day), elevated ALT (>40 U/L), obesity (normal, simple central, simple overweight, both), elevated SUA (>357 μmol/ L for women and >420 μmol/ L for men), CRP (<1, 1–3, >3 mg/L), and MetS (no/yes).

Abbreviation. CI, confidence interval; HR, hazard ratio; SD, standard deviation; IPAQ, international physical activity questionnaire; MetS, metabolic syndrome; BMI, body mass index; ALT, alanine transaminase; SUA, serum uric acid; CRP, C-reactive protein; RERI, relative excess risk due to interaction.

Interestingly, occasional snoring was still not associated with NAFLD after stratification in either population, except in participants age <45 years, and those who were mental (versus manual) labourers in Kailuan. Heterogeneous effects were detected after stratifying by age and occupation type in Kailuan (Figs. 2 and 3; Tables 3, 4, S2 and S3). The association between occasional snoring and NAFLD risk was stronger among young participants (age < 45 years), and mental labourers.

## Discussion

In this study, we established an association of self-reported snoring with NAFLD in a sampling-based cross-sectional population, and we validated this association in an independent large prospective cohort. Our findings indicated that self-reported snoring was significantly associated with a higher risk of subsequently developing NAFLD during a 10-year follow-up. These associations were independent of known risk factors for NAFLD such as obesity (defined according to BMI and WC in the present study), MetS and its components, age, smoking, sedentary behaviour, physical inactivity, elevated CRP, elevated SUA, and high salt intake^1,16,17^. To our knowledge the present study is the first to provide evidence of a direct association between snoring and an increased risk of NAFLD.

The association between habitual snoring and NAFLD was greater among lean participants (BMI < 24 or those with normal BMI and WC) in both populations. These results suggest that the presence of elevated BMI (or elevated BMI and WC) may buffer the effects of snoring on NAFLD. This is concordant with previous studies in which strong associations were found between obesity and increased prevalence and development of snoring^15^.

In stratification analyses of two independent populations, habitual snoring was still significantly associated with increased prevalence and incidence of NAFLD in each stratum after stratifying by sex, age, MetS, arterial hypertension, and BMI in both analyses. Interestingly, the risk of NAFLD was significantly associated with habitual snoring in participants who did not have hyperglycaemia, and had normal WC, in both analyses; however, this was not the case in workers who exhibited hyperglycaemia in both analyses; and had elevated WC in the cohort analyses. These results suggest that hyperglycaemia and elevated WC may be stronger risk factors for the development of NAFLD than habitual snoring.

High BMI, elevated WC, the presence of diabetes, and the presence of MetS are well-known primary risk factors for the development of NAFLD that are usually concurrent with NAFLD^1^. Lean NAFLD is also not uncommon; however, this represents a clinical challenge because the diagnosis of NAFLD may be delayed or ignored in such cases owing to an absence of the aforementioned common comorbidities^18^. Notably, the results of the present study suggest that habitual snoring may be a useful early indicator of NAFLD even in the absence of common comorbidities.

Some inconsistent results pertaining to simple central obesity and simple overweight were obtained in the two study populations. This may be owing to different distributions of body types among these populations. In Tongmei, similar proportions of participants had simple central obesity (12.0%) and simple overweight (12.7%), and the largest proportion of participants (43.1%) exhibited both forms of obesity. In Kailuan, 27.3% of participants had simple overweight and only 7.4% had simple central obesity, and the largest proportion of participants (45.5%) exhibited normal BMI and WC. Furthermore, compared with non-snorers who had normal BMI and WC, ORs and HRs of NAFLD in participants with elevated BMI and/or elevated WC were dramatically increased. This confirmed that controlling weight and WC are very important in the management of NAFLD.

The proportions of NAFLD in men and women were inconsistent in the present two populations; this conflict is common, as previously reported^19^. Interestingly, female non-snorers and male snorers had similar risks of NAFLD in both populations. In stratified analyses, however, habitual snoring was consistently significantly associated with increased risk of NAFLD among men and women. In a recently reported cross-sectional study, self-reported snoring status was compared with polysomnography results; in that study women tended to under-report their snoring and men tended to over-report snoring^20^. This apparent sex difference in self-reporting may contribute to the comparatively lower risk in men than in women.

Snoring is an early symptom of OSAS^12^, and OSAS has been incorporated in two prediction models of nonalcoholic steatohepatitis in morbidly obese patients, to optimize the selection of patients for liver biopsy^21^. The prevalence of OSAS in the general population is relatively low^30^, however, and the diagnosis of OSAS relies on polysomnography. Habitual snoring is common in the general population and can be easily detected by co-sleepers. Therefore, we speculate that self-reported habitual snoring can be incorporated in prediction models of NAFLD. This association was confirmed in the present cohort study, but needs to be validated in more extensive populations.

The mechanisms involved in the association between snoring and NAFLD have not been elucidated, but several explanations for the causal relationship between OSAS and NAFLD have been suggested^22,23^. Sleep-disordered breathing leads to chronic intermittent hypoxia, which may cause liver injury, lipid deposition, inflammation, and fibrogenesis via activation of hypoxia inducible factor, nuclear factor kappa-light-chain-enhancer of activated B cells, or the induction of endoplasmic reticulum stress, tissue inflammation, and insulin resistance^22,23^. OSAS increases the number of micro-arousals, the accumulation of which causes sleep fragmentation and reduces its restorative value^24,25^. In a randomized controlled trial, it was concluded that the sound of snoring probably increased the number of micro-arousals^26^. Collectively, these potential mechanisms may constitute the pathophysiological basis of the association between snoring and increased risk of NAFLD.

Liver biopsy is the gold standard for NAFLD diagnosis but biopsy is not feasible in a large population-based study. Ultrasound is a widely accessible imaging technique for the detection of fatty liver in clinical and population settings owing to its relatively low cost and verified safety^27^. To minimize the effects of misclassification via ultrasound in the present study, two separate analyses were conducted. In one analysis, at least two positive determinations via ultrasound were required to qualify a participant as a new NAFLD case^16^ in a separate analysis, an alternative definition of “at least one positive report” was used. Similar results were obtained using either definition.

In the present study, the presence and frequency of snoring was based on self-reporting that was undoubtedly influenced by input from participants’ families, and this may have resulted in under- or over-reporting^15,20^. Notably, snoring can be detected by co-sleepers; detection, quantification, and data acquisition using more objective methods was beyond the scope of the present study, which used data derived from very large population-based cohorts. With regard to future studies, it has been reported that low-cost no-contact or contact microphones that do not affect sleep quality are effective, and acoustic analysis of snoring is now considered a highly accurate diagnostic tool for OSAS versus polysomnography^28^. Further studies using such methods are encouraged, to confirm the findings of the present study. Effective treatments are also available for snoring, such as low-level continuous positive airway pressure (CPAP), oropharyngeal exercises, oral appliance therapy, and the use of specific types of pillows^29–33^. A recent review indicated that CPAP, the first-line treatment for OSAS, may be beneficial with regard to liver disease in people with OSAS, independent of metabolic risk factors^34^.

The two populations included in the current study were occupation-based, so caution should be used in extrapolating the results to more general populations. Compared with population-based studies, the common issue of an unbalanced ratio of men and women existed; because the majority of coal mine staff are men. Interestingly, the sex ratio in Kailuan was close to that in the general population, which was at least partly because alcohol drinkers were excluded from the analysis and the proportion of male drinkers was larger than that of female drinkers. Furthermore, interaction analysis consistently indicated that there were no significant differences in ORs or HRs between men and women.

Detailed dietary information and a history of OSAS at baseline were obtained in Tongmei, and high total energy intake was a risk factor for NAFLD in a crude model. Comparable dietary information and OSAS history were not obtained in Kailuan, however, so the two populations could not be compared in this regard. Moreover, individuals with genotype 3 HCV infection were not excluded in this study because only a history of HCV infection was collected in Tongmei and Kailuan; genotype testing was not feasible in these two large population-based studies. However, the prevalence of genotype 3 HCV infection is low in Chinese populations^11,35^. Lastly, although several potential confounders were adjusted in the models, because the current investigation was an observational study, the present results may have been affected by additional independent NAFLD risk factors and snoring risk factors that could not be incorporated into the analysis owing to unavailability, such as myopenia measured via body composition, genetic susceptibility genes, neck circumference, or cranio-facial differences; in addition, anatomical aspects such as single or multi-level obstruction, muscle tonus, and length of the upper airway may influence the intensity of snoring^1,15,36^.

## Conclusion

Snoring is a common condition that may be associated with the prevalence and 10-year incidence of NAFLD. Habitual snoring may be particularly useful as a low-cost, non-invasive, and convenient predictor of NAFLD, especially in individuals who do not exhibit common comorbidities. Further research investigating the underlying mechanisms involved in the association between snoring and NAFLD is warranted, as are prospective studies investigating the effects of attenuating snoring symptoms on NAFLD.

## Methods

### Study population

The Tongmei study was a cross-sectional health survey of staff working for Datong Coal Mine Group^37,38^ (Datong, China). Using two-stage cluster stratified sampling, 4,341 employees (84.2% men, age 18–65 years) were recruited from July 2013 to December 2013. The Kailuan study, an ongoing prospective cohort study of coal mine staff working for the Kailuan Group^16,39^ (Tangshan, China) from June 2006 to December 2017, was also used for validation. In 2006 and 2007 (baseline), 101,510 employees and retirees (80.3% men, age 18–97 years) were recruited in 11 hospitals, and then followed biennially. The designs and methods of the Tongmei and Kailuan studies have been detailed elsewhere^16,37–39^.

Both the Tongmei study and Kailuan study consisted of face-to-face interviews, clinical examinations, and acquisition of laboratory data. These studies were conducted in compliance with the Declaration of Helsinki, and the protocols were reviewed and approved by the Ethics Committees of Shanxi Medical University and Kailuan General Hospital, respectively. Written informed consent was obtained from all study participants before data collection.

Exclusion criteria included (1) self-reported alcohol consumption, or missing alcohol consumption history data; (2) liver cirrhosis; (3) presence of diseases such as OSAS, thyroid disease, or cancer; (4) taking a drug that could potentially affect snoring or NAFLD, or long-term use of sedative-hypnotic drugs; and (5) missing ultrasound data or data pertaining to other covariates. In the Kailuan study, we additionally excluded (6) participants with NAFLD at baseline; (7) participants without follow-up data; (8) participants who self-reported drinking during follow-up; and (9) participants with liver cirrhosis during follow-up.

### Data collection and definitions

Blood pressure measurement, anthropometry, overnight fasting blood specimen collection, physical examination, and abdominal ultrasound were performed in the morning by trained and certified nurses, physicians, or experienced radiologists who were blinded to the laboratory findings, in accordance with standard protocols and techniques^40^. In face-to-face interviews, each participant was asked about demographics, lifestyle, nutrition, and physical activity, and participants’ medical history was collected via self-administered questionnaires.

In the Tongmei study, physical activity level and sedentary behaviour were assessed using the International Physical Activity Questionnaire (IPAQ)^41^, and a validated semi-quantitative food frequency questionnaire was used to obtain data reflecting dietary intake in the past year^42^. Notably, no nutrition survey was involved in the Kailuan baseline survey. Laboratory staff assessed blood biochemical indexes and blood glucose using automatic analysers (Tongmei: SIEMENS ADVIA 1800 at the General Hospital of Datong Coal Mining Group; Kailuan: Hitachi 747 at the Central Laboratory of the Kailuan General Hospital). C-reactive protein and serum uric acid were only tested in Kailuan. Alcohol consumption was ascertained using a structured questionnaire, including the consumption of beer, wine, and spirits.

### Definitions and calculations

NAFLD was diagnosed by experienced radiologists via abdominal ultrasonography (Tongmei: portable MyLab 30CV, Biosound Esaote; Kailuan: HD-15, Philips) at recruitment in both studies; NAFLD was monitored biennially from 2008–2017 in Kailuan. The criteria for determination of NAFLD suggested by the Chinese Liver Disease Association were used^1^ as previously described^16^. Alternative causes, such as alcohol consumption and systemic diseases or medications before a diagnosis of NAFLD were ruled out according to the history of drinking, drug use, and diseases. Owing to the relatively low sensitivity and specificity of ultrasonography for detecting moderate or severe liver steatosis compared with histology^16,27^, NAFLD was defined as positive liver steatosis determined via ultrasonography, and incident NAFLD was defined as patients without NAFLD at baseline and with at least two reports of positive liver steatosis at any time from 2008 to 2017^16^. For cases of incident NAFLD, person-time of follow-up was calculated from the date of the 2006 survey (baseline) to the date of the first NAFLD diagnosis; for the remainder, person-time of follow-up was calculated from the date of baseline to the date of the last follow-up.

Snoring status was self-reported by participants, and was often ascertained with the assistance of family members, with regard to the question “Have you ever snored while asleep?” In both studies, there were three response choices for that question: “never”, “occasionally (1 or 2 times/week)”, and “habitually (≥3 times/week)”.

MetS was diagnosed with the presence of any three of the following five factors^1^: (1) elevated waist circumference: waist circumference >90 cm in men and >85 cm in women; (2) arterial hypertension: arterial blood pressure ≥130/85 mmHg or on antihypertensive therapy; (3) hypertriglyceridemia: fasting serum triglycerides ≥1.7 mmol/L or on lipid-lowering medication; (4) low HDL-C: fasting serum HDL-C < 1.0 mmol/L in men or <1.3 mmol/L in women; and (5) hyperglycaemia: fasting serum glucose ≥5.6 mmol/L or a history of type 2 diabetes mellitus. Obesity was defined based on both BMI and WC, and included four categories: normal (normal BMI and WC), simple central obesity (normal BMI and elevated WC), simple overweight (elevated BMI and normal WC), and both forms of obesity (elevated BMI and WC).

Physical activity and sedentary behaviour were defined as low, moderate, or high in accordance with IPAQ guidelines^41^. Total energy intake per day was calculated based on China Food Composition^30^, and categorized according to tertiles. In the Kailuan study, physical activity and sedentary behaviour were respectively evaluated based on answers to questions pertaining to the frequency of physical activity and duration of sedentary behaviour. Salt intake was self-reported as low, medium, or high as described previously^16^. Elevated serum liver enzymes was defined as any among ALT, AST, and GGT higher than the upper normal limit (40, 45, and 58 U/L, respectively). Elevated SUA was defined as >420 μmol/L in men and >357 μmol/L in women. Current smokers were those who had smoked at least one cigarette per month during the past year.

### Statistical methods

Statistical analyses were performed using SAS 9.4 (SAS Institute Inc., Cary, NC, USA). Characteristics of the study samples were summarized as frequencies and percentages according to NAFLD status and study. Logistic regression was used in cross-sectional analyses and Cox regression was used in cohort analyses, to investigate associations between snoring and NAFLD. Crude odds ratios (ORs) or crude hazard ratios (HRs) together with the corresponding 95% confidence intervals (CIs) were calculated via univariate regression. Adjusted ORs or adjusted HRs and corresponding 95% CIs were also calculated in multivariate regression, controlling for potential confounders. In view of the different covariates collected in Tongmei and Kailuan, different covariates were used. Lastly, adjustments were made in the two populations for age, sex, marital status, education, income, workplace, current tobacco smoking, BMI, physical activity, sedentary behaviour, perceived salt intake, and MetS. Additional adjustments for daily total energy intake and elevated serum liver enzymes were applied to data derived from the Tongmei study, and additional adjustments for elevated ALT, elevated SUA, and CRP were applied to data derived from the Kailuan study.

We investigated interactions on additive and multiplicative scales between snoring and sex, age, workplace, obesity, BMI, and MetS and its components, which may have modified the associations between snoring and NAFLD. Additive interaction was evaluated via relative excess risk owing to interaction (RERI) and the corresponding 95% CI^43^.

### Sensitivity analysis

Modified Poisson models were used to test the sensitivity of the results in cross-sectional analyses^44^. A broader definition of NAFLD was used to test the sensitivity of the results in cohort analyses, where incident NAFLD cases were defined as those without NAFLD at baseline and with at least one positive ultrasonography result during 2008–2017.

## Supplementary information

Supplementary information.
