# Supplementary material for: Self-reported snoring is associated with nonalcoholic fatty liver disease
Source: Sci Rep. 2020 Jun 9;10:9267. doi: 10.1038/s41598-020-66208-1 (PMC7283303; doi:10.1038/s41598-020-66208-1)
Supplement: Supplementary file 1 — Supplementary information. [file 41598_2020_66208_MOESM1_ESM.docx]

# Self-reported snoring is associated with nonalcoholic fatty liver disease

Hui Wang^1^, Qian Gao^1^, Simin He^1^, Yanping Bao^2^, Hongwei Sun^1^, Lingxian Meng^1^, Jie Liang^1^, Chenming Sun^3^, Shuohua Chen^4^, Liying Cao^5^, Wei Huang^6^, Yanmin Zhang^7^, Jianjun Huang^8^, Shouling Wu^4^* and Tong Wang^1^*

^1^Department of Epidemiology and Health Statistics, School of Public Health, Shanxi Medical University, Taiyuan 030001, China;  ^2^National Institute on Drug Dependence, Peking University, 38 Xueyuan Road, Beijing 100191, China; ^3^Department of Urology, General Hospital of Datong Coal Mining Group, Datong 037003, China; ^4^Department of Cardiology, Kailuan General Hospital, Tangshan 063000, China; ^5^Department of Hepatobiliary Surgery, Kailuan General Hospital, Tangshan 063000, China; ^6^Department of Ultrasonography, Kailuan General Hospital, Tangshan 063000, China; ^7^Department of Gastroenterology, Kailuan General Hospital, Tangshan 063000, China; ^8^Department of Neurosurgery, General Hospital of Datong Coal Mining Group, Datong 037003, China

Correspondence:

Tong Wang

Department of Epidemiology and Health Statistics, School of Public Health, Shanxi Medical University, 56 Xinjian South Rd, Taiyuan, Shanxi, 030001, China;

Tel.:+86 0351 4135397;

Fax: +86 0351 4135998;

Email: tongwang@sxmu.edu.cn

Shouling Wu

Department of Cardiology, Kailuan General Hospital, 57 Xinhua East Rd, Tangshan, Hebei, 063000, China;

Tel.: +86 315 3025655;

Fax: +86 315 3725312;

Email: drwusl@163.com

**Appendix**

# Calculation of the follow-up rate in the Kailuan cohort

The follow-up rate was calculated using the formula “(N-#without follow-up)/N”, where N is the number of participants determined to have a normal liver in ultrasound testing at baseline and who never consumed alcohol at baseline and during follow-up, that is, 101,510−32,192−1118−42,647-285=25,268 (Figure 1). We used 25,268 instead of 101,510, the number of participants at baseline (2006–2007), because the former was the number of the target population in the present study. Moreover, some individuals were excluded owing to positive liver steatosis at baseline or drinking, as well as loss to follow-up; however these individuals were not included in #without follow-up, which would result in overestimation of the follow-up rate.

# Table S1 Baseline characteristics of participants according to NAFLD status and study (Tongmei and Kailuan) (continued)

|  | **Tongmei cross-sectional population in 2013** | | | | **Kailuan cohort at baseline (2006–2007)** | | | |
| --- | --- | --- | --- | --- | --- | --- | --- | --- |
| **Characteristic** | **All^†^ (n=2153)** | **Normal liver^‡^ status(n=1515)** | **Fatty liver^‡^ status(n=638)** | **Crude OR^§^ (95% CI)** | **All^†^ (n=19587)** | **Normal liver^‡^ status(n=15011)** | **Fatty liver^‡^ status(n=4576)** | **Crude HR^¶^ (95% CI)** |
| Education |  |  |  |  |  |  |  |  |
| Illiterate/primary | 51(2.37%) | 34(66.67%) | 17(33.33%) | 1 | 1858(9.47%) | 1556(83.75%) | 302(16.25%) | 1 |
| Junior high school | 542(25.17%) | 363(66.97%) | 179(33.03%) | 0.99(0.54–1.81) | 14548(74.16%) | 10918(75.05%) | 3630(24.95%) | **1.47(1.31–1.65)** |
| Senior high school | - | - | - | **-** | 2172(11.07%) | 1673(77.03%) | 499(22.97%) | **1.32(1.14–1.52)** |
| College or higher | - | - | - | **-** | 1040(5.30%) | 891(85.67%) | 149(14.33%) | **0.77(0.64–0.94)** |
| Senior high school or college | 1307(60.71%) | 935(71.54%) | 372(28.46%) | 0.80(0.44–1.44) | - | - | - | **-** |
| Bachelor’s degree or higher | 253(11.75%) | 183(72.33%) | 70(27.67%) | 0.77(0.40–1.46) | - | - | - | **-** |
| Statistics(P value) |  | 4.65(0.1989) |  |  |  | 96.07(<.0001) |  |  |
| Monthly income |  |  |  |  |  |  |  |  |
| ≤￥4000 | 582(27.03%) | 412(70.79%) | 170(29.21%) | 1 | - | - | - | **-** |
| >￥4000–￥6000 | 911(42.31%) | 627(68.83%) | 284(31.17%) | 1.10(0.87–1.38) | - | - | - | **-** |
| >￥6000 | 660(30.65%) | 476(72.12%) | 184(27.88%) | 0.94(0.73–1.20) | - | - | - | **-** |
| Statistics(P value) |  | 2.06(0.3566) |  |  |  | - |  |  |
| Monthly income |  |  |  |  |  |  |  |  |
| <￥600 | - | - | - | **-** | 4282(21.86%) | 3314(77.39%) | 968(22.61%) | 1 |
| ￥600–￥800 | - | - | - | **-** | 13255(67.67%) | 10084(76.08%) | 3171(23.92%) | 1.04(0.97–1.12) |
| ￥800–￥1000 | - | - | - | **-** | 1149(5.87%) | 894(77.81%) | 255(22.19%) | 0.97(0.85–1.12) |
| >￥1000 | - | - | - | **-** | 901(4.60%) | 719(79.80%) | 182(20.20%) | 0.88(0.75–1.03) |
| Statistics(P value) |  |  |  |  |  | 6.73(0.0810) |  |  |
| Workplace |  | - |  |  |  |  |  |  |
| Underground | 1078(50.07%) | 763(70.78%) | 315(29.22%) | 1 | 3132(15.99%) | 2503(79.92%) | 629(20.08%) | 1 |
| Surface | 1075(49.93%) | 752(69.95%) | 323(30.05%) | 1.04(0.86–1.25) | 16455(84.01%) | 12508(76.01%) | 3947(23.99%) | **1.15(1.06–1.25)** |
| Statistics(P value) |  | 0.18(0.6748) |  |  |  | 10.97(0.0009) |  |  |
| Occupation type |  |  |  |  |  |  |  |  |
| Mental labour | 566(26.52%) | 399(70.49%) | 167(29.51%) | 1 | 1405(7.17%) | 1113(79.22%) | 292(20.78%) | 1 |
| Physical labour | - | - | - | **-** | 18182(92.83%) | 13898(76.44%) | 4284(23.56%) | **1.18(1.05–1.32)** |
| Light physical labour | 1070(50.14%) | 728(68.04%) | 342(31.96%) | 1.12(0.90–1.40) |  |  |  |  |
| Heavy physical labour | 498(23.34%) | 371(74.50%) | 127(25.50%) | 0.82(0.62–1.07) |  |  |  |  |
| Statistics(P value) |  | 6.81(0.0332) |  |  |  | 7.23(0.0072) |  |  |
| Perceived salt intake |  |  |  |  |  |  |  |  |
| Low | 545(25.31%) | 381(69.91%) | 164(30.09%) | 1 | 1489(7.60%) | 1180(79.25%) | 309(20.75%) | 1 |
| Medium | 1000(46.45%) | 712(71.20%) | 288(28.80%) | 0.94(0.75–1.18) | 16841(85.98%) | 12887(76.52%) | 3954(23.48%) | **1.13(1.01–1.27)** |
| High | 608(28.24%) | 422(69.41%) | 186(30.59%) | 1.02(0.80–1.32) | 1257(6.42%) | 944(75.10%) | 313(24.90%) | **1.25(1.07–1.47)** |
| Statistics(P value) |  | 0.66(0.7204) |  |  |  | 7.96(0.0187) |  |  |
| Sedentary |  |  |  |  |  |  |  |  |
| Low | 650(30.19%) | 456(70.15%) | 194(29.85%) | 1 | - | - | - | **-** |
| Moderate | 766(35.58%) | 516(67.36%) | 250(32.64%) | 1.14(0.91–1.43) | - | - | - | **-** |
| High | 737(34.23%) | 543(73.68%) | 194(26.32%) | 0.84(0.66–1.06) | - | - | - | **-** |
| Statistics(P value) |  | 7.20(0.0273) |  |  |  | - |  |  |
| Sedentary duration per day |  |  |  |  |  |  |  |  |
| <4 Hours | - | - | - | **-** | 15860(80.97%) | 12083(76.19%) | 3777(23.81%) | 1 |
| 4–8 Hours | - | - | - | **-** | 3240(16.54%) | 2537(78.30%) | 703(21.70%) | **0.92(0.84–0.99)** |
| >8 Hours | - | - | - | **-** | 487(2.49%) | 391(80.29%) | 96(19.71%) | **0.81(0.66–0.99)** |
| Statistics(P value) |  | - |  |  |  | 8.45(0.0146) |  |  |
| Degree of IPAQ |  |  |  |  |  |  |  |  |
| Low | 186(8.64%) | 134(72.04%) | 52(27.96%) | 1 | - | - | - | **-** |
| Moderate | 633(29.40%) | 441(69.67%) | 192(30.33%) | 1.12(0.78–1.61) | - | - | - | **-** |
| High | 1334(61.96%) | 940(70.46%) | 394(29.54%) | 1.08(0.77–1.52) | - | - | - | **-** |
| Statistics(P value) |  | 0.40(0.8167) |  |  |  | - |  |  |
| Physical activity |  |  |  |  |  |  |  |  |
| No | - | - | - | **-** | 984(5.02%) | 766(77.85%) | 218(22.15%) | 1 |
| Occasional | - | - | - | **-** | 15892(81.14%) | 12137(76.37%) | 3755(23.63%) | 1.07(0.94–1.23) |
| Always | - | - | - | **-** | 2711(13.84%) | 2108(77.76%) | 603(22.24%) | 1.04(0.89–1.21) |
| Statistics(P value) |  | - |  |  |  | 1.57(0.4555) |  |  |
| Total energy intake per day |  |  |  |  |  |  |  |  |
| Low | 759(35.25%) | 554(72.99%) | 205(27.01%) | 1 | - | - | - | **-** |
| Moderate | 700(32.51%) | 508(72.57%) | 192(27.43%) | 1.02(0.81–1.29) | - | - | - | **-** |
| High | 694(32.23%) | 453(65.27%) | 241(34.73%) | **1.44(1.15–1.80)** | - | - | - | **-** |
| Statistics(P value) |  | 12.77(0.0017) |  |  |  | - |  |  |
| Elevated serum liver enzymes |  |  |  |  |  |  |  |  |
| No | 1534(71.25%) | 1212(79.01%) | 322(20.99%) | 1 | - | - | - | **-** |
| Yes | 619(28.75%) | 303(48.95%) | 316(51.05%) | **3.93(3.21–4.79)** | - | - | - | **-** |
| Statistics(P value) |  | 191.11(<.0001) |  |  |  | - |  |  |
| Elevated ALT |  |  |  |  |  |  |  |  |
| No | 1620(75.24%) | 1260(77.78%) | 360(22.22%) | 1 | 19061(97.31%) | 14632(76.76%) | 4429(23.24%) | 1 |
| Yes | 533(24.76%) | 255(47.84%) | 278(52.16%) | **3.82(3.10–4.69)** | 526(2.69%) | 379(72.05%) | 147(27.95%) | **1.26(1.07–1.48)** |
| Statistics(P value) |  | 172.35(<.0001) |  |  |  | 7.54(0.0060) |  |  |
| Elevated AST |  |  |  |  |  |  |  |  |
| No | 2047(95.08%) | 1465(71.57%) | 582(28.43%) | 1 | - | - | - | **-** |
| Yes | 106(4.92%) | 50(47.17%) | 56(52.83%) | **2.82(1.90–4.18)** | - | - | - | **-** |
| Statistics(P value) |  | 28.77(<.0001) |  |  |  | - |  |  |
| Elevated GGT |  |  |  |  |  |  |  |  |
| No | 1917(89.20%) | 1407(73.40%) | 510(26.60%) | 1 | - | - | - | **-** |
| Yes | 232(10.80%) | 104(44.83%) | 128(55.17%) | **3.40(2.57–4.48)** | - | - | - | **-** |
| Statistics(P value) |  | 80.92(<.0001) |  |  |  | - |  |  |
| Elevated SUA |  |  |  |  |  |  |  |  |
| No | - | - | - | **-** | 18859(96.28%) | 14483(76.80%) | 4376(23.20%) | 1 |
| Yes | - | - | - | **-** | 728(3.72%) | 528(72.53%) | 200(27.47%) | **1.31(1.14–1.51)** |
| Statistics(P value) |  | - |  |  |  | 13.87(0.0002) |  |  |
| CRP (mg/L) |  |  |  |  |  |  |  |  |
| <1 | - | - | - | **-** | 11888(60.69%) | 9325(78.44%) | 2563(21.56%) | 1 |
| 1–3 | - | - | - | **-** | 4509(23.02%) | 3354(74.38%) | 1155(25.62%) | **1.28(1.19–1.37)** |
| >3 | - | - | - | **-** | 3190(16.29%) | 2332(73.10%) | 858(26.90%) | **1.38(1.27–1.49)** |
| Statistics(P value) |  | - |  |  |  | 88.30(<.0001) |  |  |

^†^Values are displayed as number (column percent) for categorical variables and mean (SD) for continuous variables.
^‡^Values are displayed as number (row percent) for categorical variables and mean (SD) for continuous variables.
^§^Logistic regression was used for calculating crude ORs in univariate analyses; The ORs according to BMI were calculated using Firth's penalized likelihood because of quasi-complete separation of data points.
^¶^ Cox regression was used for calculating crude HRs in univariate analyses.
**Abbreviation**. CI confidence interval; OR odds ratio; HR, hazard ratio; SD, standard deviation; IPAQ, international physical activity questionnaire; ALT, alanine transaminase; AST, aspartate aminotransferase; GGT, gamma glutamyl transpeptidase; SUA, serum uric acid; CRP, C-reactive protein; NAFLD, non-alcoholic fatty liver disease.

# Table S2 Effect modification of snoring on NAFLD in Tongmei: (OR (95% CI), P value (continued)

|  | | **Occasional snorers (N=774) vs. Non–snorers (N=879)** | | | **Habitual snorers (N=500) vs. Non–snorers (N=879)** | | |
| --- | --- | --- | --- | --- | --- | --- | --- |
| **Variables** | **Snoring** | **NAFLD (No/Yes)** | **OR (95% CI), P value** | **Stratified OR  (95% CI), P value** | **NAFLD (No/Yes)** | **OR** **(95% CI), P value** | **Stratified OR  (95% CI), P value** |
| Workplace^†^ |  |  |  |  |  |  |  |
| Underground | No | 340/79 | 1(ref) | 1(ref) | 340/79 | 1(ref) | 1(ref) |
| Underground | Yes | 284/110 | 1.38(0.90–2.11), P=0.1381 | 1.38(0.90–2.11), P=0.1381 | 139/126 | **1.99(1.29–3.06), P=0.0020** | **1.99(1.29–3.06), P=0.0020** |
| Surface | No | 367/93 | 1.16(0.68–1.96), P=0.5909 | 1(ref) | 367/93 | 1.09(0.64–1.85), P=0.7586 | 1(ref) |
| Surface | Yes | 269/111 | 1.29(0.77–2.16), P=0.3287 | 1.12(0.74–1.69), P=0.5967 | 116/119 | **2.36(1.40–3.96), P=0.0013** | **2.17(1.40–3.36), P=0.0005** |
| Ratio of ORs |  |  | 0.81(0.45–1.46), P=0.4859 |  |  | 1.09(0.59–2.01), P=0.7779 |  |
| RERI |  |  | –0.24(–0.99~0.50), P=0.5241 |  |  | 0.28(–0.76~1.32), P=0.5926 |  |
| Occupation type^†^ |  |  |  |  |  |  |  |
| Mental | No | 192/45 | 1(ref) | 1(ref) | 192/45 | 1(ref) | 1(ref) |
| Mental | Yes | 143/62 | 1.23(0.69–2.18), P=0.4817 | 1.23(0.69–2.18), P=0.4817 | 64/60 | **2.41(1.31–4.45), P=0.0049** | **2.41(1.31–4.45), P=0.0049** |
| Light physical | No | 334/92 | 0.91(0.51–1.62), P=0.7503 | 1(ref) | 334/92 | 1.14(0.65–2.00), P=0.6552 | 1(ref) |
| Light physical | Yes | 266/117 | 1.19(0.68–2.08), P=0.5535 | 1.30(0.86–1.97), P=0.2147 | 128/133 | **1.96(1.10–3.49), P=0.0217** | **1.73(1.13–2.63), P=0.0110** |
| Heavy physical | No | 173/35 | 0.64(0.32–1.29), P=0.2095 | 1(ref) | 173/35 | 0.95(0.47–1.91), P=0.8754 | 1(ref) |
| Heavy physical | Yes | 137/40 | 0.71(0.36–1.42), P=0.3374 | 1.12(0.60–2.09), P=0.7250 | 61/52 | **2.54(1.23–5.24), P=0.0118** | **2.69(1.41–5.11), P=0.0026** |
| Ratio of ORs |  |  | 1.06(0.52–2.14), P=0.8735 | Light physical vs mental |  | 0.72(0.34–1.49), P=0.3693 | Light physical vs mental |
| Ratio of ORs |  |  | 0.91(0.39–2.12), P=0.8273 | Heavy physical vs mental |  | 1.11(0.46–2.70), P=0.8131 | Heavy physical vs mental |
| RERI |  |  | 0.05(–0.71~0.80), P=0.9059 | Light physical vs mental |  | –0.59(–2.00~0.83), P=0.4165 | Light physical vs mental |
| RERI |  |  | –0.15(–0.95~0.64), P=0.7065 | Heavy physical vs mental |  | 0.18(–1.39~1.75), P=0.8220 | Heavy physical vs mental |
| Arterial hypertension ^‡^ |  |  |  |  |  |  |  |
| No | No | 444/80 | 1(ref) | 1(ref) | 444/80 | 1(ref) | 1(ref) |
| No | Yes | 349/88 | 0.98(0.63–1.53), P=0.9179 | 0.98(0.63–1.53), P=0.9179 | 133/93 | **1.76(1.10–2.81), P=0.0182** | **1.76(1.10–2.81), P=0.0182** |
| Yes | No | 263/92 | 1.25(0.79–1.97), P=0.3431 | 1(ref) | 263/92 | 1.26(0.81–1.95), P=0.3078 | 1(ref) |
| Yes | Yes | 204/133 | 1.52(0.98–2.36), P=0.0644 | 1.21(0.77–1.91), P=0.4002 | 122/152 | **2.27(1.44–3.58), P=0.0004** | **1.81(1.16–2.82), P=0.0094** |
| Ratio of ORs |  |  | 1.24(0.66–2.34), P=0.4989 |  |  | 1.03(0.54–1.94), P=0.9318 |  |
| RERI |  |  | 0.29(–0.44~1.02), P=0.4325 |  |  | 0.26(–0.78~1.29), P=0.6259 |  |
| Hyperglycaemia ^‡^ |  |  |  |  |  |  |  |
| No | No | 643/133 | 1(ref) | 1(ref) | 643/133 | 1(ref) | 1(ref) |
| No | Yes | 497/172 | 1.04(0.74–1.47), P=0.8292 | 1.04(0.74–1.47), P=0.8292 | 209/180 | **1.93(1.34–2.77), P=0.0004** | **1.93(1.34–2.77), P=0.0004** |
| Yes | No | 62/36 | 1.53(0.81–2.89), P=0.1893 | 1(ref) | 62/36 | 1.54(0.83–2.85), P=0.1688 | 1(ref) |
| Yes | Yes | 47/46 | **2.17(1.16–4.06), P=0.0154** | 1.42(0.63–3.20), P=0.4031 | 44/63 | **1.94(1.11–3.39), P=0.0207** | 1.26(0.60–2.66), P=0.5488 |
| Ratio of ORs |  |  | 1.36(0.56–3.29), P=0.4902 |  |  | 0.65(0.29–1.48), P=0.3083 |  |
| RERI |  |  | 0.60(–0.95~2.15), P=0.4481 |  |  | –0.53(–1.96~0.89), P=0.4638 |  |
| Hypertriglyceridemia^‡^ |  |  |  |  |  |  |  |
| No | No | 542/80 | 1(ref) | 1(ref) | 542/80 | 1(ref) | 1(ref) |
| No | Yes | 417/94 | 0.92(0.60–1.43), P=0.7138 | 0.92(0.60–1.43), P=0.7138 | 174/78 | 1.40(0.89–2.21), P=0.1436 | 1.40(0.89–2.21), P=0.1436 |
| Yes | No | 165/92 | 1.47(0.92–2.35), P=0.1028 | 1(ref) | 165/92 | 1.53(0.98–2.39), P=0.0642 | 1(ref) |
| Yes | Yes | 136/127 | **1.93(1.22–3.07), P=0.0052** | 1.31(0.82–2.08), P=0.2526 | 81/167 | **3.49(2.18–5.57), P<.0001** | **2.28(1.44–3.63), P=0.0005** |
| Ratio of ORs |  |  | 1.42(0.76–2.67), P=0.2747 |  |  | 1.63(0.86–3.09), P=0.1351 |  |
| RERI |  |  | 0.54(–0.31~1.39), P=0.2163 |  |  | **1.56(0.19~2.92), P=0.0254** |  |
| Low HDL–C ^‡^ |  |  |  |  |  |  |  |
| No | No | 320/40 | 1(ref) | 1(ref) | 320/40 | 1(ref) | 1(ref) |
| No | Yes | 224/44 | 1.09(0.58–2.04), P=0.7873 | 1.09(0.58–2.04), P=0.7873 | 85/34 | 1.54(0.81–2.93), P=0.1866 | 1.54(0.81–2.93), P=0.1866 |
| Yes | No | 387/132 | 1.19(0.71–2.02), P=0.5081 | 1(ref) | 387/132 | 1.49(0.90–2.47), P=0.1178 | 1(ref) |
| Yes | Yes | 329/177 | 1.30(0.77–2.19), P=0.3313 | 1.09(0.75–1.57), P=0.6588 | 170/211 | **2.79(1.65–4.72), P=0.0001** | **1.87(1.29–2.72), P=0.0011** |
| Ratio of ORs |  |  | 1.00(0.49–2.05), P=0.9932 |  |  | 1.21(0.58–2.52), P=0.6048 |  |
| RERI |  |  | 0.01(–0.78~0.80), P=0.9735 |  |  | 0.76(–0.38~1.90), P=0.1928 |  |
| Elevated WC^‡^ |  |  |  |  |  |  |  |
| No | No | 455/26 | 1(ref) | 1(ref) | 455/26 | 1(ref) | 1(ref) |
| No | Yes | 315/31 | 1.25(0.66–2.36), P=0.4993 | 1.25(0.66–2.36), P=0.4993 | 112/28 | **2.12(1.09–4.13), P=0.0277** | **2.12(1.09–4.13), P=0.0277** |
| Yes | No | 252/146 | **2.71(1.59–4.64), P=0.0003** | 1(ref) | 252/146 | **3.37(2.01–5.66), P<.0001** | 1(ref) |
| Yes | Yes | 238/190 | **2.82(1.65–4.82), P=0.0001** | 1.04(0.72–1.50), P=0.8319 | 143/217 | **5.71(3.34–9.79), P<.0001** | **1.69(1.17–2.45), P=0.0053** |
| Ratio of ORs |  |  | 0.83(0.40–1.74), P=0.6285 |  |  | 0.80(0.38–1.70), P=0.5626 |  |
| RERI |  |  | –0.14(–1.40~1.12), P=0.8312 |  |  | 1.22(–0.84~3.29), P=0.2448 |  |

^†^ adjusted for age (<45 or ≥45 years), sex, marital status (single, married, divorced/widowed/separated), education (illiterate/primary, junior high school, senior high school or college, bachelor’s degree or higher), income (≤4000, >4000–6000, >6000 RMB), workplace (underground/surface), occupation type (mental labour/light physical labour/heavy physical labour), current tobacco smoking (yes, no), perceived salt intake (low, medium, high), degree of IPAQ (low, moderate, high), degree of sedentary (low, moderate, high), total energy intake per day (low, moderate, high), elevated serum liver enzymes (no/yes), obesity (normal, central, overweight, both), and MetS (no/yes).
^‡^ adjusted for age (<45 years or ≥45 years), sex, marital status (single, married, divorced/widowed/separated), education (illiterate/primary, junior high school, senior high school or college, bachelor or higher), income (≤4000, >4000–6000, >6000 RMB), workplace (underground/surface), occupation type (mental labour/light physical labour/heavy physical labour), current tobacco smoking (yes, no), perceived salt intake (low, medium, high), degree of IPAQ (low, moderate, high), degree of sedentary (low, moderate, high), total energy intake per day (low, moderate, high), elevated serum liver enzymes (no/yes), BMI (<24, 24–<28, ≥28 kg/m^2^), arterial hypertension (no/yes), hypertriglyceridemia (no/yes), hypertriglyceridemia (no/yes), low HDL-C (no/yes), and elevated WC (no/yes).

**Abbreviation**. CI confidence interval; OR odds ratio; HR, hazard ratio; SD, standard deviation; IPAQ, international physical activity questionnaire; MetS, metabolic syndrome; HDL-C, high-density lipoprotein cholesterol; BMI, body mass index; WC, waist circumference; RERI, relative excess risk due to interaction.

# Table S3 Effect modification of snoring on NAFLD in Kailuan: (OR (95% CI), P value (continued)

|  | | **Occasional snorers (N=2944) vs. Non-snorers (N=15128)** | | | **Habitual snorers (N=1515) vs. Non-snorers (N=15128)** | | |
| --- | --- | --- | --- | --- | --- | --- | --- |
| **Variables** | **Snoring** | **NAFLD (No/Yes)** | **HR (95% CI), P value** | **Stratified HR  (95% CI), P value** | **NAFLD (No/Yes)** | **HR (95% CI), P value** | **Stratified HR  (95% CI), P value** |
| Workplace^†^ |  |  |  |  |  |  |  |
| Underground | No | 1558/375 | 1(ref) | 1(ref) | 1558/375 | 1(ref) | 1(ref) |
| Underground | Yes | 597/131 | 0.92(0.75–1.12), P=0.3958 | 0.92(0.75–1.12), P=0.3958 | 348/123 | 1.18(0.96–1.45), P=0.1187 | 1.18(0.96–1.45), P=0.1187 |
| Surface | No | 10093/3102 | 0.94(0.83–1.06), P=0.3230 | 1(ref) | 10093/3102 | 0.95(0.84–1.08), P=0.4491 | 1(ref) |
| Surface | Yes | 1701/515 | 0.97(0.84–1.13), P=0.7187 | 1.04(0.94–1.15), P=0.4983 | 714/330 | **1.29(1.10–1.51), P=0.0017** | **1.35(1.19–1.53), P<.0001** |
| Ratio of HRs |  |  | 1.13(0.91–1.41), P=0.2801 |  |  | 1.15(0.91–1.45), P=0.2550 |  |
| RERI |  |  | 0.12(-0.09~0.32), P=0.2611 |  |  | 0.16(-0.11~0.42), P=0.2561 |  |
| Occupation type^†^ |  |  |  |  |  |  |  |
| Physical | No | 10950/3338 | 1(ref) | 1(ref) | 10950/3338 | 1(ref) | 1(ref) |
| Physical | Yes | 1998/540 | 0.95(0.86–1.05), P=0.3096 | 0.95(0.86–1.05), P=0.3096 | 950/406 | **1.28(1.14–1.43), P<.0001** | **1.28(1.14–1.43), P<.0001** |
| Mental | No | 701/139 | 0.86(0.71–1.03), P=0.1000 | 1(ref) | 701/139 | 0.89(0.74–1.08), P=0.2327 | 1 |
| Mental | Yes | 300/106 | **1.36(1.10–1.68), P=0.0040** | **1.59(1.24–2.05), P=0.0003** | 112/47 | **1.44(1.07–1.95), P=0.0171** | **1.62(1.16–2.25),P=0.0048** |
| Ratio of HRs |  |  | **1.68(1.28-2.20), P=0.0002** |  |  | 1.27(0.89-1.80), P=0.1869 |  |
| RERI |  |  | **0.56(0.25~0.87), P=0.0004** |  |  | 0.27(-0.19~0.73), P=0.2455 |  |
| Arterial hypertension^‡^ |  |  |  |  |  |  |  |
| No | No | 6130/1619 | 1(ref) | 1(ref) | 6130/1619 | 1(ref) | 1(ref) |
| No | Yes | 1258/319 | 1.07(0.94–1.21), P=0.3130 | 1.07(0.94–1.21), P=0.3130 | 425/166 | **1.32(1.12–1.55), P=0.0011** | **1.32(1.12–1.55), P=0.0011** |
| Yes | No | 5521/1858 | **1.17(1.09–1.26), P<.0001** | 1(ref) | 5521/1858 | **1.18(1.09–1.26), P<.0001** | 1(ref) |
| Yes | Yes | 1040/327 | 1.09(0.96–1.24), P=0.2006 | 0.93(0.82–1.05), P=0.2288 | 637/287 | **1.45(1.26–1.67), P<.0001** | **1.23(1.08–1.41), P=0.0021** |
| Ratio of HRs |  |  | 0.87(0.73–1.03), P=0.1027 |  |  | 0.94(0.76–1.15), P=0.5258 |  |
| RERI |  |  | -0.15(-0.34~0.03), P=0.1044 |  |  | -0.04(-0.32~0.23), P=0.7579 |  |
| Hyperglycaemia ^‡^ |  |  |  |  |  |  |  |
| No | No | 9209/2700 | 1(ref) | 1(ref) | 9209/2700 | 1(ref) | 1(ref) |
| No | Yes | 1739/490 | 1.01(0.91–1.12), P=0.8355 | 1.01(0.91–1.12), P=0.8355 | 767/341 | **1.37(1.21–1.54), P<.0001** | **1.37(1.21–1.54), P<.0001** |
| Yes | No | 2442/777 | 1.02(0.94–1.11), P=0.6460 | 1(ref) | 2442/777 | 1.01(0.94–1.10), P=0.7297 | 1(ref) |
| Yes | Yes | 559/156 | 0.95(0.81–1.13), P=0.5737 | 0.94(0.78–1.12), P=0.4567 | 295/112 | 1.04(0.85–1.26), P=0.7157 | 1.02(0.83–1.25), P=0.8315 |
| Ratio of HRs |  |  | 0.92(0.76–1.13), P=0.4394 |  |  | **0.75(0.60–0.94), P=0.0130** |  |
| RERI |  |  | -0.08(-0.27~0.12), P=0.4346 |  |  | **-0.34(-0.60~-0.09), P=0.0089** |  |
| Hypertriglyceridemia^‡^ |  |  |  |  |  |  |  |
| No | No | 9563/2414 | 1(ref) | 1(ref) | 9563/2414 | 1(ref) | 1(ref) |
| No | Yes | 1770/418 | 1.03(0.92–1.15), P=0.6436 | 1.03(0.92–1.15), P=0.6436 | 760/268 | **1.31(1.14–1.50), P<.0001** | **1.31(1.14–1.50), P<.0001** |
| Yes | No | 2088/1063 | **1.57(1.46–1.69), P<.0001** | 1(ref) | 2088/1063 | **1.59(1.48–1.72), P<.0001** | 1(ref) |
| Yes | Yes | 528/228 | **1.46(1.27–1.69), P<.0001** | 0.93(0.80–1.08), P=0.3527 | 302/185 | **1.92(1.63–2.25), P<.0001** | **1.20(1.02–1.42), P=0.0272** |
| Ratio of HRs |  |  | 0.91(0.76–1.08), P=0.2877 |  |  | 0.92(0.75–1.12), P=0.4125 |  |
| RERI |  |  | -0.13(-0.37~0.11), P=0.2772 |  |  | 0.01(-0.32~0.35), P=0.9338 |  |
| Low HDL-C ^‡^ |  |  |  |  |  |  |  |
| No | No | 10224/2985 | 1(ref) | 1(ref) | 10224/2985 | 1(ref) | 1(ref) |
| No | Yes | 2024/563 | 1.00(0.91–1.10), P=0.9945 | 1.00(0.91–1.10), P=0.9945 | 948/392 | **1.25(1.11–1.40), P=0.0002** | **1.25(1.11–1.40), P=0.0002** |
| Yes | No | 1427/492 | 0.94(0.85–1.04), P=0.2424 | 1(ref) | 1427/492 | 0.95(0.86–1.05), P=0.2934 | 1(ref) |
| Yes | Yes | 274/83 | 0.89(0.71–1.11), P=0.3058 | 0.94(0.74–1.20), P=0.6306 | 114/61 | **1.31(1.01–1.70), P=0.0403** | **1.38(1.05–1.81), P=0.0193** |
| Ratio of HRs |  |  | 0.94(0.73–1.21), P=0.6511 |  |  | 1.11(0.83–1.48), P=0.4863 |  |
| RERI |  |  | -0.05(-0.28~0.18), P=0.6501 |  |  | 0.11(-0.25~0.48), P=0.5379 |  |
| Elevated WC^‡^ |  |  |  |  |  |  |  |
| No | No | 8918/2121 | 1(ref) | 1(ref) | 8918/2121 | 1(ref) | 1(ref) |
| No | Yes | 1802/384 | 0.98(0.87–1.09), P=0.6775 | 0.98(0.87–1.09), P=0.6775 | 775/259 | **1.35(1.18–1.54), P<.0001** | **1.35(1.18–1.54), P<.0001** |
| Yes | No | 2733/1356 | **1.30(1.21–1.40), P<.0001** | 1(ref) | 2733/1356 | **1.30(1.21–1.40), P<.0001** | 1(ref) |
| Yes | Yes | 496/262 | **1.32(1.15–1.52), P<.0001** | 1.02(0.89–1.17), P=0.8042 | 287/194 | **1.52(1.29–1.78), P<.0001** | 1.16(0.99–1.36), P=0.0636 |
| Ratio of HRs |  |  | 1.04(0.88–1.24), P=0.6347 |  |  | 0.86(0.71–1.05), P=0.1505 |  |
| RERI |  |  | 0.05(-0.16~0.25), P=0.6553 |  |  | -0.13(-0.42~0.15), P=0.3561 |  |

^†^ adjusted for age (<45, 45–<55, 55–<65, ≥65 years), sex, marital status (single, married, divorced/widowed/separated), education (illiterate/primary, junior high school, senior high school, college or higher), income (<600, 600–800, 800–1000, >1000 Yuan RMB), workplace (underground/surface), occupation type (mental labour/physical labour), current tobacco smoking (yes, no), perceived salt intake (low, medium, high), physical activity (no, occasional, always), sedentary duration (<4, 4–8, >8 hours per day), elevated ALT (>40 U/L), obesity (normal, simple central, simple overweight, both), elevated SUA (>357μmol/ L for female and >420μmol/ L for male), CRP (<1, 1–3, >3 mg/L), and MetS (No/Yes).
^‡^ adjusted for age (<45, 45–<55, 55–<65, ≥65 years), sex, marital status (single, married, divorced/widowed/separated), education (illiterate/primary, junior high school, senior high school, college or higher), income (<600, 600–800, 800–1000, >1000 Yuan RMB), workplace (underground/surface), occupation type (mental labour/physical labour), current tobacco smoking (yes, no), perceived salt intake (low, medium, high), physical activity (no, occasional, always), sedentary duration (<4, 4–8, >8 hours per day), elevated ALT (>40 U/L), BMI (<18, 18–<24, 24–<28, ≥28 kg/m^2^), elevated SUA (>357μmol/ L for female and >420μmol/ L for male), CRP (<1, 1–3, >3 mg/L), arterial hypertension (no/yes), hypertriglyceridemia (no/yes), hypertriglyceridemia (no/yes), low HDL-C (no/yes), and elevated WC (no/yes).
**Abbreviation**. CI, confidence interval; HR, hazard ratio; SD, standard deviation; IPAQ, international physical activity questionnaire HDL-C, high-density lipoprotein cholesterol; BMI, body mass index; WC, waist circumference; ALT, alanine transaminase; SUA, serum uric acid; CRP, C-reactive protein; RERI, relative excess risk due to interaction.
